# Prognostic Model Based on Sex, ALBI Grade, and ALR in Intermediate-to-Advanced HCC Patients Receiving Targeted Therapy Combined with ICIs and Interventional Treatment

**DOI:** 10.3390/cancers18132063

**Published:** 2026-06-25

**Authors:** Xiaomeng Hu, Huiying Yan, Siyi Li, Zhiqiang Han, Hua Li, Xi Wei, Wei Zhang, Huikai Li

**Affiliations:** 1 Department of Ultrasound Diagnosis and Treatment, Tianjin Medical University Cancer Institute & Hospital, National Clinical Research Center for Cancer, Key Laboratory of Cancer Prevention and Therapy, Tianjin’s Clinical Research Center for Cancer, Tianjin 300060, China; huxiaomeng2201@tjmuch.com (X.H.); weixi@tjmuch.com (X.W.); 2 Liver Cancer Center, Tianjin Medical University Cancer Institute & Hospital, National Clinical Research Center for Cancer, Key Laboratory of Cancer Prevention and Therapy, Tianjin’s Clinical Research Center for Cancer, Tianjin 300060, China; yanhuiying@tmu.edu.cn; 3 Department of Tumor Cell Biology, Tianjin Medical University Cancer Institute & Hospital, National Clinical Research Center for Cancer, Key Laboratory of Cancer Prevention and Therapy, Tianjin’s Clinical Research Center for Cancer, Tianjin 300060, China; lisiyi0415@tmu.edu.cn; 4 Department of Anesthesiology, Tianjin Medical University Cancer Institute & Hospital, National Clinical Research Center for Cancer, Key Laboratory of Cancer Prevention and Therapy, Tianjin’s Clinical Research Center for Cancer, Tianjin 300060, China; hanzhiqiang@tmu.edu.cn; 5 Department of Endoscopy, Tianjin Medical University Cancer Institute & Hospital, National Clinical Research Center for Cancer, Key Laboratory of Cancer Prevention and Therapy, Tianjin’s Clinical Research Center for Cancer, Tianjin 300060, China; lihua@tjmuch.com

**Keywords:** HCC, triple therapy, prognostic model, ALBI grade, ALR

## Abstract

**Background/Objectives**: Triple therapy combining targeted therapy (lenvatinib/bevacizumab), immune checkpoint inhibitors (ICIs), and interventional therapy (TACE/HAIC) has shown promising efficacy, but clinical outcomes differ among patients. We developed and tested a prognostic model based on sex, ALBI grade, and ALR to estimate survival in patients with intermediate-to-advanced HCC receiving triple therapy. **Methods**: This single center retrospective study included 184 intermediate-to-advanced HCC patients between November 2017 and December 2024. The patients enrolled received lenvatinib (*n* = 88) or bevacizumab (*n* = 96) plus PD-1/PD-L1 inhibitors and interventional therapy. The risk scoring model was derived from univariate Cox regression, LASSO Cox regression, and multivariate Cox regression analyses that were screened for independent prognostic factors. The median risk score defined the cutoff for separating patients into two risk subgroups (high- and low-risk). Overall survival (OS) across subgroups was evaluated and compared by Kaplan–Meier analysis and log-rank test. Model performance was evaluated using the C-index, time-dependent AUC at 6, 12, and 24 months, calibration curves, the Brier score, and decision curve analysis, with internal validation performed via Bootstrap resampling. **Results**: Multivariate analysis identified male sex, ALBI grade 3, and a high ALR level as independent risk factors of poorer OS. The resulting risk model showed a C-index of 0.62. Moreover, the median OS differed significantly between the two risk groups (*p* < 0.001). The model displayed moderate discrimination, with AUCs of 0.66, 0.66, and 0.74 at 6, 12, and 24 months. Calibration and the Brier score showed reasonable agreement and acceptable prediction errors. No interaction between risk factors and treatment type was observed (*p* > 0.05), indicating model applicability to both lenvatinib and bevacizumab-based regimens. **Conclusions**: A prognostic model integrating sex, ALBI grade, and ALR can offer some capacity to stratify survival risk in intermediate-to-advanced HCC patients. However, its overall discriminative performance is limited, and further validation in larger and external cohorts is needed to confirm its clinical value.

## Simple Summary

Hepatocellular carcinoma (HCC) continues to be a major driver of global cancer mortality. For patients diagnosed at intermediate-to-advanced stages, therapeutic choices are frequently constrained. Although the triple therapy integrated with immunotherapy, interventional procedures and targeted therapy have demonstrated promising efficacy, patients still showed significant differences in heterogeneity. A straightforward prognostic model was constructed and tested in this study, relying on three clinical parameters: sex, ALBI grade, and ALR. Data from 184 patients were examined, leading to the observation that this model effectively differentiates patients with favorable outcomes from those at higher risk after combination therapy. Given that all component variables are collected in clinical practice, this tool can help clinicians perform risk stratification and facilitate more personalized treatment decisions for patients with intermediate-to-advanced HCC.

## 1. Introduction

Hepatocellular carcinoma (HCC) is the third leading contributor to global cancer mortality, characterized by high malignancy [1]. Tyrosine kinase inhibitors (TKIs) have long been the primary first-line approach for unresectable advanced HCC [2]. Combination strategies that include ICIs plus anti-angiogenic agents [3], TKIs [4], or other ICIs [5] have demonstrated superior survival benefits over conventional targeted monotherapy and are gradually becoming the new standard of first-line treatment [6].

Concurrently, combining locoregional therapies like TACE and HAIC with systemic treatments could improve conversion rates and survival in intermediate-to-advanced HCC (BCLC B/C) [7]. Studies indicate that TACE can enhance tumor immune responses, which helps boost the antitumor efficacy of ICIs [8,9]. The underlying mechanism is that TACE induces local ischemic necrosis to enhance the therapeutic response to subsequent systemic therapy [10]. Multiple studies have shown the clinical benefits of therapy combined with TACE and systemic treatment [11,12]. The phase III LAUNCH trial also confirmed the effectiveness of combining locoregional and systemic therapy [13].

In this context, the combination of immunotherapy, targeted therapy, and interventional procedures is attracting growing interest because early-phase studies show high ORR and strong translational potential [14]. For example, a phase II trial found ORRs ranging from 56.7% to 74.4% with lenvatinib or bevacizumab plus a PD-1 inhibitor and TACE [15,16]. A real-world study supports this benefit: triple therapy significantly improves outcomes compared with TACE alone [17].

Although triple therapy is a promising new strategy for intermediate-to-advanced HCC, much of the relevant evidence comes from small-sample Phase II studies or real-world cohorts, and the ORR does not completely reflect long-term survival benefits. In addition, the pretreatment risk stratification remains a challenge because of the considerable heterogeneity among patients. Most existing HCC prognostic models were developed in patients receiving monotherapy or no anticancer treatment; whether these models are applicable to patients treated with triple therapy remains unclear. Accordingly, we retrospectively analyzed a cohort of patients with unresectable HCC who received triple therapy. Using clinical data from this cohort, we constructed and validated a model to predict OS, aiming to provide an exploratory basis for risk stratification and individualized treatment in this population.

## 2. Materials and Methods

### 2.1. Patient Cohort

We retrospectively analyzed clinicopathological data from 184 HCC patients treated between November 2017 and December 2024. Inclusion criteria: (1) HCC diagnosed according to AASLD clinical or pathological criteria [18]; (2) CNLC stage IIb, IIIa, or IIIb (corresponding to BCLC stage B or C); (3) first-line triple therapy based on lenvatinib or bevacizumab; (4) a clearly defined treatment start date, complete baseline candidate variables, and follow-up information suitable for OS analysis. Exclusion criteria: (1) another malignancy; (2) interventional therapies other than TACE or HAIC; (3) systemic regimens other than PD 1/PD L1 antibodies; (4) patients with missing data on key baseline variables, treatment start date, or OS outcomes. Patients enrolled were assigned to lenvatinib or bevacizumab subgroup based on the treatment regimen received. The study protocol was approved by the Ethics Committee of Tianjin Medical University Cancer Hospital. All recipients along with their families provided written consent following a complete explanation of the study.

### 2.2. Statistical Analysis

All statistical analyses were conducted using R software (version 4.2.2), with a two-sided *p* value of less than 0.05 considered statistically significant. Data analysis and visualization were mainly carried out using the survival, survminer, glmnet, timeROC, rms, Hmisc and car.

The distribution of continuous variables was assessed using Shapiro–Wilk or Kolmogorov–Smirnov test. Variables following a normal distribution were summarized as mean ± standard deviation, with intergroup comparisons performed via Student’s *t*-test. For those not normally distributed, data were presented as median and interquartile range, and group differences were evaluated with the Mann–Whitney U test. Categorical variables were summarized as frequencies and percentages, and intergroup comparisons performed using the chi-square test (χ^2^ test) or Fisher’s exact test.

## 3. Results

### 3.1. Baseline Characteristics

This study comprised 184 patients treated with triple therapy between November 2017 and December 2024. The regimen combined targeted therapy with PD-1/PD-L1 inhibitors and interventional therapy. The mean age of the patients was 57.5 years (95% CI, 50.0–65.0), and the majority were male (83.7%). Most patients were HBV-positive (84.8%), had an ECOG PS of 0 (77.2%), and were classified as Child–Pugh A (79.9%). According to CNLC staging, 15.8% of patients were stage IIb, 28.3% were stage IIIa, and 56.0% were stage IIIb. Most patients (88.0%) underwent fewer than six interventional treatment sessions. By May 2025, follow-up data were available for 184 patients who had received triple therapy. During the follow-up period, 77 deaths were recorded, including 38 in the lenvatinib group and 39 in the bevacizumab group. A total of 107 patients were censored, with a censoring rate of 58.2%. The median follow-up time estimated using the reverse Kaplan–Meier method was 20.4 months (17.4–24.3 months). Among patients who remained alive at the end of follow-up, the shortest follow-up duration was 3 months. All baseline candidate variables included in the statistical analyses had complete data and no imputation for missing values was performed (Table 1).

### 3.2. Variable Selection and Prognostic Factor Analysis

**Figure 1:**
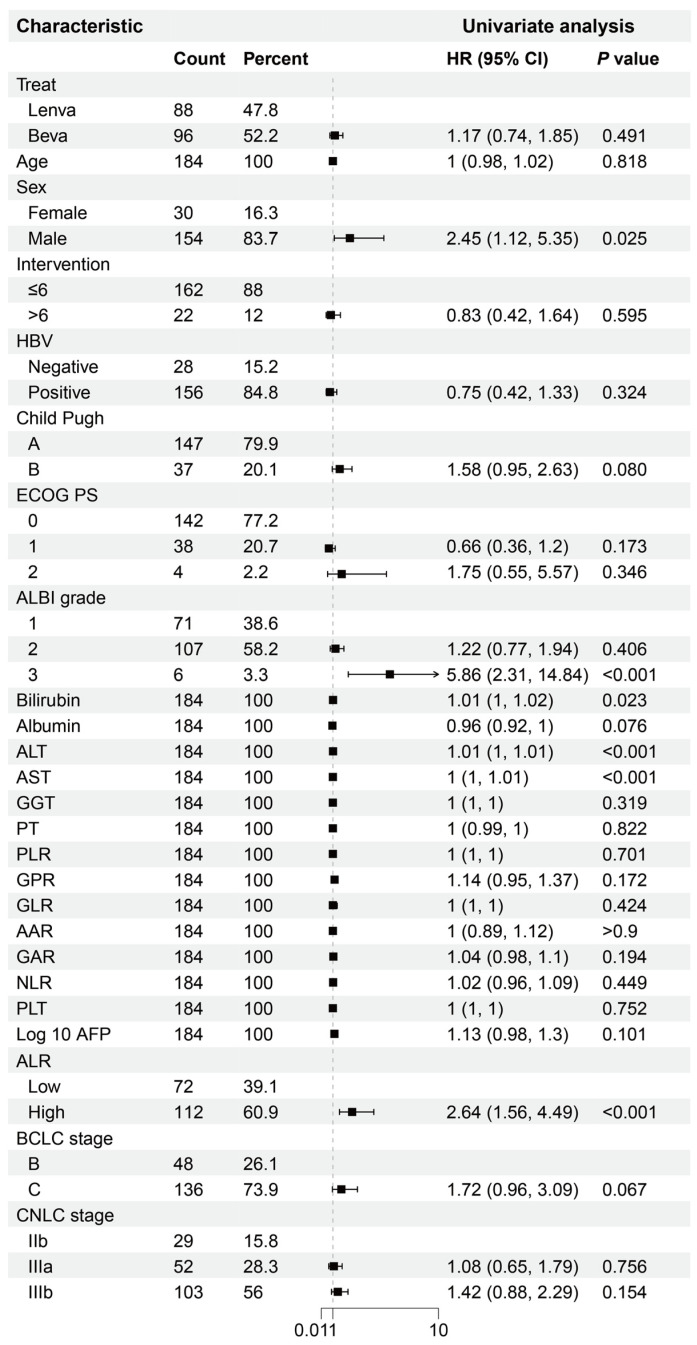
The Cox regression analysis is for OS.

**Figure 2:**
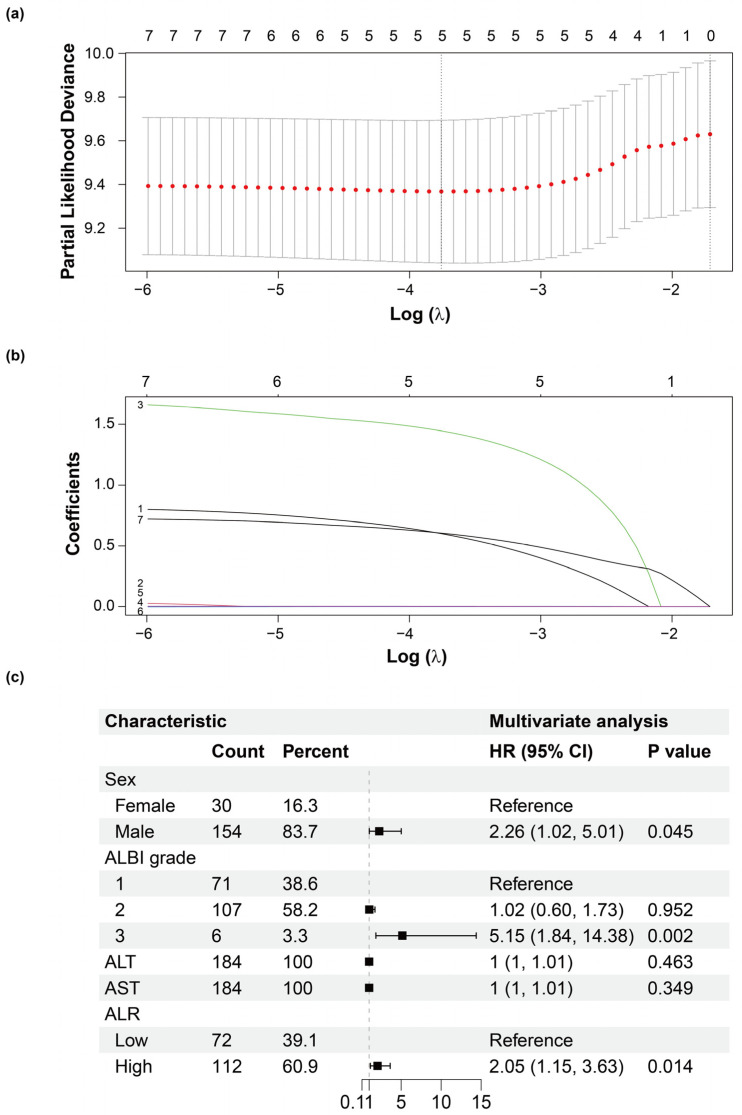
Identification of prognostic factors using LASSO Cox regression and subsequent multivariable Cox regression analysis. (**a**) Cross-validation curve for selecting the optimal penalty parameter λ in the LASSO Cox regression model. The vertical dotted lines represent λmin and λ1se, and the numbers above the plot indicate the number of nonzero coefficients. (**b**) LASSO coefficient profiles of candidate prognostic variables according to log(λ). (**c**) Forest plot of the multivariable Cox regression model constructed using variables selected by LASSO Cox regression. Hazard ratios and 95% confidence intervals are shown. HR > 1 indicates an increased risk of death.

Univariate analysis identified sex, ALBI grade, bilirubin, AST, ALT, and ALR as significant factors affecting the prognosis of HCC (all *p* < 0.05). Treatment regimen, age, number of interventional sessions, HBV status, Child–Pugh class, CNLC stage, albumin, GGT, PT, and other factors were not significant (*p* > 0.05) (Figure 1). To reduce the risk of overfitting caused by an excessive number of candidate variables, those with a univariate *p* value < 0.05 were subsequently entered into a LASSO Cox regression model for variable selection. Ten-fold cross-validation served to determine the optimal value of the regularization parameter λ. The cross-validation curve indicated that the model achieved the minimum cross-validated error when λ was set to λmin (Figure 2a). The LASSO coefficient path plot demonstrated that as λ increased, the coefficients of the candidate variables gradually shrank toward zero (Figure 2b). At λmin, the regression coefficients for sex, ALBI grade, ALR, ALT, and AST remained nonzero and were therefore incorporated into the subsequent multivariate Cox analysis.

**Figure 3:**
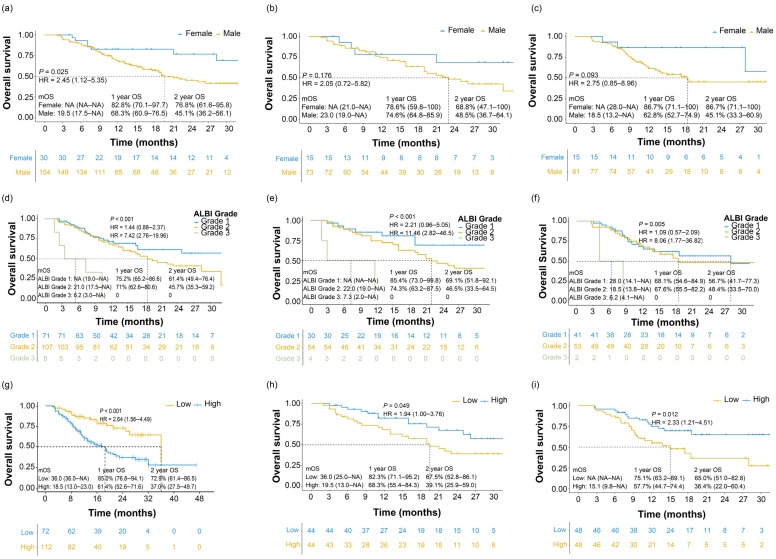
Kaplan–Meier survival curves for three independent prognostic factors. Overall survival (OS) was stratified by sex (**a**–**c**), ALBI grade (**d**–**f**), and ALR level (**g**–**i**) in the entire cohort (**a**,**d**,**g**), the lenvatinib treatment group (**b**,**e**,**h**), and the bevacizumab treatment group (**c**,**f**,**i**).

Multivariate analysis confirmed that male sex, ALBI grade 3, and high ALR level were independent risk factors for survival and prognosis (*p* < 0.05). A higher mortality risk was observed for male patients than for female patients, with a HR of 2.26 (95% CI: 1.02–5.01, *p* < 0.05). Compared with patients with ALBI grade 1, those with ALBI grade 3 showed a significantly elevated risk of death (HR = 5.15, 95% CI: 1.84–14.38, *p* < 0.05). Patients in the high-ALR group faced an increased risk of death compared with those in the low-ALR group (HR = 2.05, 95% CI: 1.15–3.63, *p* < 0.05) (Figure 2c). Accordingly, sex, ALBI grade, and ALR were incorporated into the final model. Based on the variables identified by multivariate analysis, Kaplan–Meier survival curves were generated for the lenvatinib group, the bevacizumab group, and the overall cohort to visually illustrate the survival differences (Figure 3).

### 3.3. Development and Validation of the Prognostic Model and Risk Classification

Using above multivariate analysis results, we established a prognostic model for survival based on sex, ALBI grade, and ALR, and calculated the coefficients for the risk score formula: Risk Score = (0.816 × male) + (0.088 × ALBI Level 2) + (1.731 × ALBI Level 3) + (0.867 × ALR high group). Specifically, male sex and the high-ALR group were each assigned a value of 1, and 0 otherwise. For ALBI grade, with grade 1 serving as the reference, dummy variables were created for grade 2 and grade 3, each taking a value of 1 when the corresponding grade was present and 0 otherwise. It should be noted that the coefficient for ALBI grade 2 was small and did not reach statistical significance in the multivariate Cox model, suggesting that its contribution to the risk score is limited compared to ALBI Grade 1. This variable was retained mainly to preserve the full three-level categorical structure of ALBI grade, rather than to interpret ALBI grade 2 as an independent risk factor on its own. Furthermore, because few patients presented with ALBI grade 3, the sample sizes and death event distributions across ALBI grades are provided as supplementary data. A higher proportion of death events was observed in the ALBI grade 3 subgroup; however, due to the small sample size, the effect estimates should be interpreted with caution (Supplemental Table S1).

**Figure 4:**
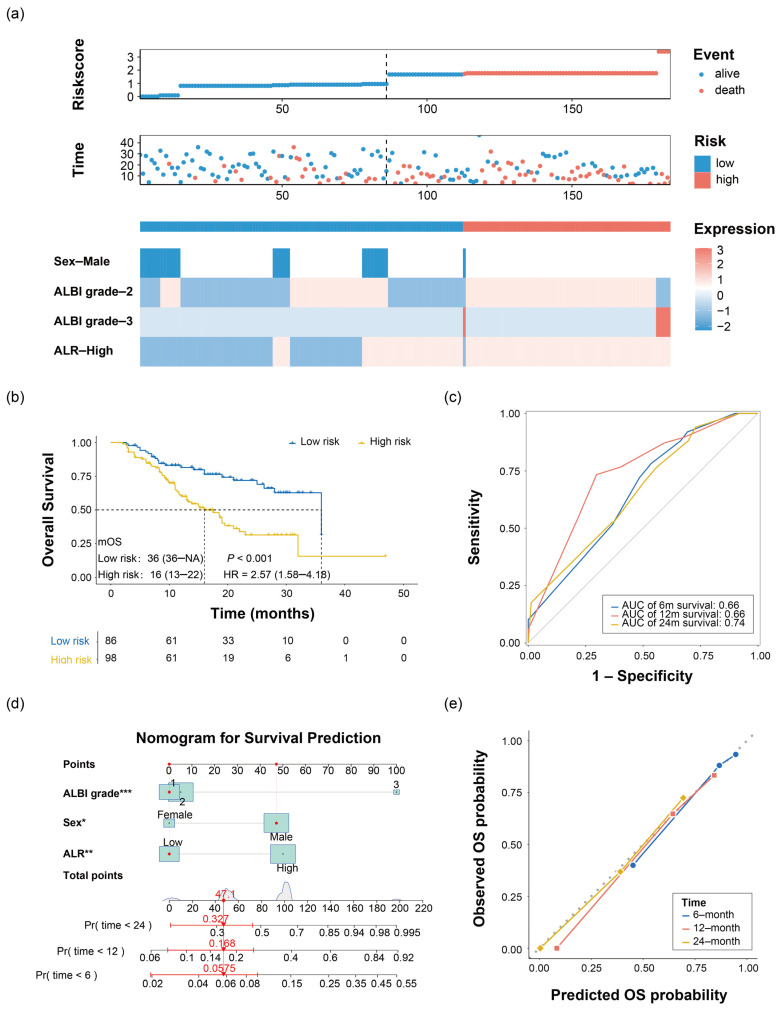
Construction and validation of the prognostic prediction model. (**a**) Patients were ranked according to their prognostic risk score, with the corresponding distribution of survival status, survival time, and key predictors, including sex, ALBI grade, and pretreatment ALR. (**b**) Kaplan–Meier survival curves comparing overall survival between the low-risk and high-risk groups (*p* < 0.001). The dashed horizontal line indicates 50% survival probability, and the dashed vertical lines indicate the corresponding median overall survival times for each group. (**c**) Time-dependent ROC curves of the risk score for predicting 6-, 12-, and 24-month OS. (**d**) Nomogram based on sex, ALBI grade, and pretreatment ALR for individualized OS prediction. For example, a male patient with ALBI grade 3 and high pretreatment ALR had a risk score of 3.414, higher than the cohort median of 1.683, and was classified as high risk; the nomogram estimated his 12-month OS probability as 8.4%. (**e**) Calibration curves for predicting 6-, 12-, and 24-month OS. The dashed diagonal line represents the ideal calibration line, where the predicted OS probability equals the observed OS probability. Statistical significance: * *p* < 0.05, ** *p* < 0.01, *** *p* < 0.001.

We then stratified all patients into low-risk subgroups (*n* = 86) and high-risk subgroups (*n* = 98), with the median risk score serving as the cutoff. The heatmap showed differences in the distribution of male sex, ALBI grade 2, ALBI grade 3, and high ALR level in comparing the two groups (Figure 4a). With a rising risk score, the proportion of patient deaths also increased. Kaplan–Meier analysis suggested a marked difference in survival between the two subgroups, where patients had significantly shorter OS in high-risk subgroup (*p* < 0.001) (Figure 4b). We then used the model to estimate long-term survival probabilities of patients. Analysis showed that the model achieved AUCs of 0.66, 0.66, and 0.74 for predicting survival at 6, 12, and 24 months, respectively, using time-dependent ROC (Figure 4c). The apparent Harrell’s C-index of the model was 0.637 (95% CI: 0.573–0.701). After internal validation using 1000 Bootstrap resamples, the optimism-corrected C-index was 0.620, indicating that the model possessed a certain degree of discriminative ability.

Using the above model, we developed a nomogram to predict survival for HCC patients which incorporates risk scores and relevant clinical variables (Figure 4d). Calibration curves indicated strong concordance between predicted and observed survival at 6, 12, and 24 months (Figure 4e). Quantitative calibration analysis revealed a global slope of 1.000 (0.645–1.355). The calibration slopes at fixed time points of 6, 12, and 24 months were 0.973, 1.027, and 1.323, respectively. Combined with the Brier score results, these findings suggest that the model’s calibration was generally acceptable and that the prediction error falls within an acceptable range. Detailed results are provided in Supplemental Table S2.

The DCA analysis revealed that this line graph shows a greater net benefit than either the treat-all or treat-none approach within clinically meaningful probability thresholds, particularly for predicting 24-month OS. These findings suggest that the model has certain clinical utility (Supplemental Figure S1).

### 3.4. Model Assumption Testing and Stability Analysis of Predictor Effects

To evaluate the validity and stability of the final Cox model, this study further conducted diagnostics for multicollinearity, tests of the proportional hazards assumption, and Bootstrap resampling analysis. The results showed that the adjusted GVIF values for all variables in the model were significantly below the preset threshold of five, indicating the absence of significant multicollinearity. The Schoenfeld residual test showed a global *p* value of 0.71, and none of the individual variable tests reached statistical significance, suggesting that the final Cox model did not show any significant violation of the proportional hazards assumption (Supplemental Table S3). Stability of the estimated effects of predictors was then assessed using 1000 Bootstrap resamples. The results showed that the direction of effects for male sex, ALBI grade 3, and the high-ALR group remained generally consistent with those in the original model, suggesting reasonable stability of the effect estimates for the main predictors (Supplemental Table S4).

To evaluate the potential impact of the small sample size of patients with ALBI grade 3 on effect estimates, we performed sensitivity analyses using Firth penalized Cox regression and Ridge Cox regression. The results showed that the direction of the effect for ALBI grade 3 was generally consistent across different modeling approaches, suggesting a certain degree of robustness. However, given the limited sample size in this subgroup, the effect estimates should still be interpreted with caution (Supplemental Table S5).

### 3.5. Comparison with Existing Models

To evaluate the predictive value of the model, we compared it with existing HCC prognostic models and inflammation-related indicators, including the Child–Pugh grade, ALBI grade, PALBI score, NLR, PLR, SII, and ALR. The comparison was based on Harrell’s C-index and time-dependent AUC at 6, 12, and 24 months (Supplemental Figure S2, Supplemental Table S6). The results showed that our prognostic model consistently exhibited higher C-index and time-dependent AUC values than any single established HCC prognostic model or inflammation-related index, indicating superior discriminative ability. Moreover, compared with ALR model, it showed improved discrimination, suggesting that combining sex, ALBI grade, and ALR provides more comprehensive prognostic information. These results indicate that the model developed in this study offers a certain predictive gain compared to existing single-indicator tools.

### 3.6. Subgroup Analysis

**Figure 5:**
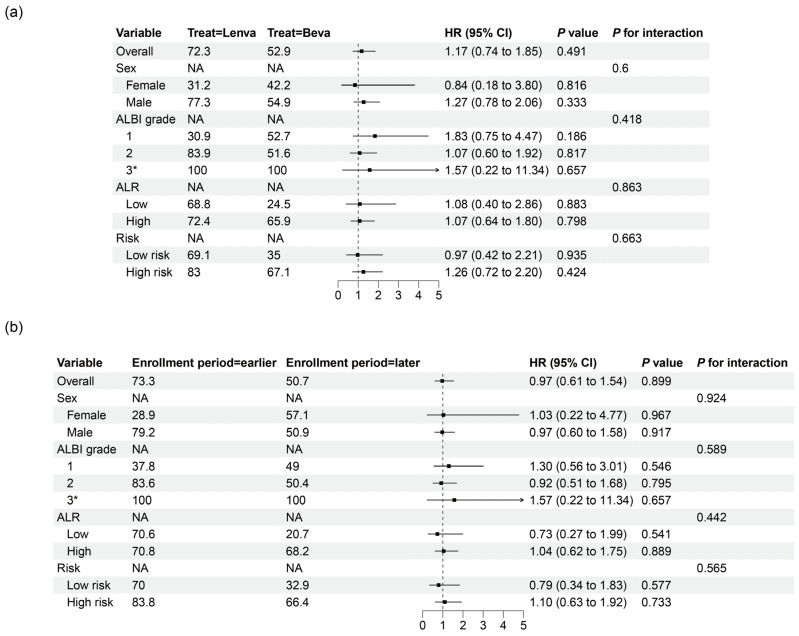
Forest plots of subgroup analyses for overall survival. (**a**) Subgroup analysis comparing overall survival between the lenvatinib-based and bevacizumab-based treatment groups across predefined clinical strata, including sex, ALBI grade, pretreatment ALR level, and prognostic risk group. (**b**) Subgroup analysis comparing overall survival between patients enrolled in the earlier and later study periods across the same strata. Hazard ratios (HRs), 95% confidence intervals (CIs), *p* values, and *p* values for interaction are shown. The vertical dashed line indicates HR = 1. No significant interactions were observed across the examined subgroups. * The ALBI grade 3 subgroup included only 6 patients, with 4 in the lenvatinib-based group and 2 in the bevacizumab-based group; therefore, the HR estimates for this subgroup should be interpreted with caution.

To investigate the consistency of the association between treatment regimen and OS across different clinical subgroups, subgroup analyses were performed according to sex, ALBI grade, ALR level, and risk stratification. The results showed that differences in OS between the lenvatinib and bevacizumab regimens were not statistically significant in the overall population or in any of the subgroups examined. Moreover, no significant interactions were observed between the treatment regimen and any of the stratification variables, indicating no substantial heterogeneity across subgroups. Given that this study was retrospective, these results should be considered exploratory and not as a basis for comparing the efficacy of the two treatment regimens. (Figure 5a).

Given the long enrollment period of this study and the possibility that systemic treatment strategies and clinical practices may have changed over time, patients were further divided into early-enrollment and late-enrollment groups based on the median enrollment time to assess the potential impact of enrollment period on model predictions. Interaction analysis revealed no significant interaction between the model’s risk stratification and enrollment period, suggesting that the prognostic stratification effect of the model was not substantially heterogeneous across enrollment-period subgroups (Figure 5b). When enrollment period was subsequently included as an adjustment covariate in the Cox model, the risk of death remained significantly elevated in the high-risk group compared with the low-risk group, with effect estimates consistent with those of the unadjusted model. This indicates that enrollment period has a limited impact on the model’s risk stratification effect. Collectively, these findings support the stability of our model across different enrollment periods. Detailed results are presented in Supplemental Table S7.

## 4. Discussion

Hepatocellular carcinoma ranks among the leading malignant tumors globally, in terms of both incidence and mortality. Because early symptoms are subtle, most patients are diagnosed at intermediate or advanced stages. About 60% are BCLC stage B or C, and lose the chance for curative resection [19]. In recent years, ICIs have dramatically changed the therapeutic landscape for advanced HCC. Some patients can achieve significant tumor regression or even become eligible for resection, which helps prolong survival [20,21]. Although ICIs have improved outcomes, monotherapy yields modest ORR and resistance is common [22]. Therefore, combining ICIs with targeted agents has gradually emerged as a key treatment approach. Although this strategy shows some clinical benefits, its ORR and long-term survival need further improvement [23,24]. Triple therapy integrating systemic and locoregional treatments has shown high ORR in early studies [16,25]. A longer PFS was reported with TACE plus bevacizumab and durvalumab in the EM-ERALD-1 trial than with TACE alone [26]. In addition, several studies, including LEAP-012 [27] and TALENT-TACE [28], have confirmed the synergistic effects of immunotherapy, targeted therapy, and interventional therapy.

Triple therapy can significantly improve treatment response and survival, but the outcomes remain heterogeneous. Some patients achieve long-term survival or even become surgical candidates, while others progress rapidly. This variability makes it difficult to identify potential responders before treatment and delays intervention for high-risk patients. Hence, clinicians urgently need a simple, reliable tool to stratify prognosis for patients on this treatment.

Reliable prognostic markers should reflect a disease’s core biology. The development of HCC depends heavily on the inflammation of liver, the characteristics of the immune microenvironment, and the level of functional reserve [29]. ALR (AST-to-lymphocyte ratio) captures hepatocyte injury, systemic inflammation and immune status. AST indicates poor prognosis in HCC because of its sensitivity to liver damage [30,31]. In contrast, antitumor immunity relies on the function of lymphocytes; a low count often indicates impaired immunity. Lymphocytes inhibit tumor proliferation and metastasis by mediating cytotoxic responses and releasing cytokines [32,33]. Studies have shown the relationship between the prognosis of HCC patient and the degree of lymphocyte infiltration in tumor tissue [34,35]. As a non-invasive index, ALR correlates with prognosis in patients with cirrhosis, liver fibrosis, and HCC [36,37,38].

Similarly, ALBI grade, based on albumin and bilirubin levels, more objectively reflects liver functional reserve [39,40]. The ALBI grade does not rely on subjective assessments compared with the Child–Pugh score. This makes it more useful for evaluating liver function and predicting HCC prognosis [41]. ALBI grade serves as a prognostic indicator for patients with cirrhosis and HCC [42,43]. Patients who received lenvatinib or atezolizumab plus bevacizumab treatment have a higher ALBI grade associated with significantly reduced survival [42,44,45].

In addition, male sex was identified as an important risk factor for survival in patients with HCC. A multicenter cohort study reported significantly longer median survival in female patients than in males (2.5 ± 2.9 years vs. 2.2 ± 2.7 years, *p* = 0.0031), and women more often presented with earlier TNM stages at diagnosis [46]. The biological basis of this survival advantage may be related to sex hormone differences. Androgens can promote hepatocyte proliferation and the formation of an inflammatory microenvironment, thereby accelerating tumor progression. In contrast, estrogens exert protective effects by inhibiting pro-inflammatory factors such as IL-6 and modulating immune surveillance. In addition, higher rates of alcohol consumption and HBV infection in the male population further aggravate the severity of underlying liver disease. Consequently, male patients are considered a high-risk group in HCC prognosis assessment and individualized treatment decisions.

It should be noted that the discriminative ability of our model remains moderate. The apparent C-index and AUC of the nomogram indicate that the model possesses some risk stratification capability, but not sufficient to guide clinical decisions on its own. Instead, it is more suitable as an exploratory prognostic tool based on routine clinical parameters. The limited predictive performance may be related to the high heterogeneity of patients. Prognosis of patients is not only influenced by liver function, inflammatory status, and host factors but also by tumor burden, vascular invasion, molecular features, the immune microenvironment, and treatment response. Our model includes only three pretreatment routine variables, thus resulting in a relatively limited scope of information.

Despite its limited discrimination, calibration and clinical net benefit analyses still suggest certain value. Calibration curves, the Brier score, and quantitative calibration indicators indicated that the model’s predictions were generally consistent with observed outcomes, with prediction errors within an acceptable range. DCA analysis revealed that it may serve as an adjunct tool for pretreatment risk stratification, follow-up management, and clinical trial design.

Compared with existing prognostic indicators, this model has certain specificity. Current HCC prognostic systems primarily focus on a single dimension (e.g., liver function or inflammatory status) and were not specifically developed in patients receiving triple therapy with ICIs, targeted therapy, and interventional therapy. Our model was built specifically on a triple therapy cohort and integrates host factors, liver function reserve, and inflammatory or immune status. In comparison with traditional scores and inflammatory indices, our model showed a numerically higher C-index and AUC, suggesting that it may provide additional prognostic information for this particular treatment population. However, given its overall modest discriminative ability and lack of external validation, this value needs further confirmation.

Subgroup and sensitivity analyses indicated reasonable stability of the model’s prognostic effect. After stratification by treatment regimen, patients at high risk in both the lenvatinib and bevacizumab groups had significantly shorter OS, and no significant interaction was observed between the risk score and treatment regimen, suggesting consistent performance of the model across different triple-therapy regimens. In addition, because the study enrolled patients over a long period, the time windows for the application of different treatment regimens did not completely overlap, raising the possibility of treatment-era bias. Sensitivity analysis using enrollment period showed that after adjusting for enrollment period, the association between risk group and OS remained largely stable, indicating that the predictive value of the model was not primarily driven by differences in enrollment era. Nevertheless, residual confounding due to changes in subsequent therapies, drug availability, and clinical management over different years cannot be fully excluded in a retrospective study.

Several limitations should be acknowledged. As a retrospective study, the design may introduce selection bias and lack an independent external validation cohort. Despite the use of LASSO screening and Bootstrap internal validation, some optimistic bias in model performance may remain. The sample size is relatively small, especially for female patients and those with ALBI grade 3, which may have led to unstable effect estimates for male sex and ALBI grade 3. Thus, these results should be interpreted with caution. The model includes only pretreatment routine variables and does not integrate information on tumor burden, vascular invasion, radiomics, circulating tumor DNA, tumor genomics, or the immune microenvironment, making it difficult to fully capture the heterogeneity of intermediate-to-advanced HCC. Our findings need validation in larger, prospective, multicenter cohorts in future studies, and further model optimization should integrate imaging features, molecular biomarkers, and dynamic treatment response indicators.

In conclusion, the prognostic model comprising ALR, ALBI grade, and sex can help stratify patients for future clinical trials of triple therapy in different risk groups. Although its discriminative ability remains moderate and insufficient for independent clinical decision-making, as a simple exploratory risk stratification tool based on routine parameters, it may provide a reference for pretreatment prognostic assessment, follow-up management, and the design of future clinical trials focusing on risk stratification.

## 5. Conclusions

In conclusion, this study demonstrates that sex, ALBI grade, and ALR are associated with OS in patients with intermediate-to-advanced HCC patients receiving triple therapy. The model constructed using these factors provides a certain ability to stratify patients by survival risk, but its C-index and AUC indicate only modest predictive performance, which is insufficient to serve as an independent clinical decision-making tool. Exploratory subgroup and interaction analyses did not reveal significant interactions between the risk score and targeted therapy regimens or treatment timing. However, due to the baseline imbalances across different treatment subgroups and the prolonged enrollment period during which treatment strategies and subsequent management may have evolved, these results should be interpreted with caution and further validated in external cohorts. Future efforts to improve model performance should integrate radiomics features, molecular biomarkers, and characteristics of the tumor immune microenvironment.

## Figures and Tables

**Table 1:** Baseline characteristics.

Variable	Overall (*n* = 184)
Age, years	57.5 (50.0, 65.0)
Gender	
Female	30 (16.3)
Male	154 (83.7)
HBV	
Negative	28 (15.2)
Positive	156 (84.8)
Intervention	
<6 times	162 (88.0)
>6 times	22 (12.0)
ECOG PS	
0	142 (77.2)
1	38 (20.7)
2	4 (2.2)
ALBI grade	
Grade 1	71 (38.6)
Grade 2	107 (58.2)
Grade 3	6 (3.3)
Albumin	38.8 (34.9, 41.3)
Bilirubin	17.1 (11.9, 24.2)
ALT	33.5 (20.8, 51.0)
AST	49.5 (30.8, 79.0)
GGT	134.0 (61.0, 243.2)
PT	12.8 (12.0, 13.9)
PLR	125.8 (91.6, 182.5)
PLT	161.0 (107.8, 226.0)
GPR	0.8 (0.4, 1.6)
GLR	108.2 (51.7, 199.8)
ALR	42.8 (23.5, 77.5)
AAR	1.5 (1.0, 2.1)
GAR	3.5 (1.6, 6.1)
NLR	2.9 (2.1, 4.2)
Log 10 AFP	2.3 (1.1, 3.7)
BCLC stage	
B	48 (26.1)
C	136 (73.9)
CNLC stage	
IIb	29 (15.8)
IIIa	52 (28.3)
IIIb	103 (56.0)
ALR group	
Low	72 (39)
High	112 (61)
Child–Pugh	
A	147 (79.9)
B	37 (20.1)

## Data Availability

The data presented in this study are available on request from the corresponding authors. The data are not publicly available due to privacy restrictions.

## Supplementary Materials

The following supporting information can be downloaded at: https://www.mdpi.com/article/10.3390/cancers18132063/s1, Figure S1: Decision curve analysis (DCA) of the nomogram for overall survival prediction. Decision curve analyses were conducted for 6-month (**a**), 12-month (**b**), and 24-month (**c**) OS prediction. The nomogram demonstrated greater net benefit than the treat-all and treat-none strategies over selected ranges of threshold probabilities, with the most pronounced clinical utility observed for 24-month OS prediction; Figure S2: Time-dependent ROC analysis of the model for overall survival prediction. Time-dependent ROC analysis was performed to evaluate the predictive performance of the proposed model with Child-Pugh grade, ALBI grade, PALBI score, NLR, PLR, SII, and ALR alone for 6-month (**a**), 12-month (**b**), and 24-month (**c**) overall survival. The proposed model demonstrated the highest area under the curve (AUC) at each time point; Table S1: Distribution of patients and death events according to ALBI grade; Table S2: Quantitative performance of the prognostic nomogram; Table S3: Multicollinearity diagnostics and proportional hazards assumption tests for the final Cox model; Table S4: Bootstrap sensitivity analysis of hazard ratio estimates in the multivariable Cox model; Table S5: Sensitivity analyses using conventional Cox, Firth penalized Cox, and ridge Cox regression; Table S6: Comparison of discriminative performance of the proposed model and established prognostic indicators; Table S7: Sensitivity analysis adjusted for enrollment period.

## Abbreviations

The following abbreviations are used in this manuscript:

ALBI	Albumin-bilirubin grade
PALBI	Platelet-albumin-bilirubin
ALR	Aspartate aminotransferase to lymphocyte ratio
TACE	Transcatheter arterial chemoembolization
HAIC	Hepatic arterial infusion chemotherapy
SMD	Standardized mean difference
IQR	Interquartile range
HBV	Hepatitis B virus
ECOG	Eastern Cooperative Oncology Group
PLR	Platelet to lymphocyte ratio
GPR	Gamma glutamyl transferase to platelet ratio
GLR	Gamma glutamyl transferase to lymphocyte ratio
AAR	Aspartate aminotransferase to alanine aminotransferase ratio
GAR	Gamma glutamyl transferase to albumin ratio
NLR	Neutrophil to lymphocyte ratio
BCLC	Barcelona Clinic Liver Cancer
CNLC	China Liver Cancer Staging
AFP	Alpha-fetoprotein
HR	Hazard Ratio
CI	Confidence Interval
SII	Systemic immune-inflammation index
C-index	Concordance index
AUC	Area under the curve
