# Supplementary material for: Prognostic Model Based on Sex, ALBI Grade, and ALR in Intermediate-to-Advanced HCC Patients Receiving Targeted Therapy Combined with ICIs and Interventional Treatment
Source: Cancers (Basel). 2026 Jun 25;18(13):2063. doi: 10.3390/cancers18132063 (PMC13359942; doi:10.3390/cancers18132063)
Supplement: Supplementary file 1 [file cancers-18-02063-s001.zip › cancers-4327304-supplementary.pdf]

Materials and Methods

1.1 Treatment Strategies

Patients received first-line triple therapy comprising targeted therapy combined with PD-1/PD-L1 antibodies and interventional therapies (TACE or HAIC). In this study, patients in the lenvatinib group received treatment from November 2017 to April 2022, while those in the bevacizumab group were treated from May 2021 to December 2024. All treatment decisions were made by a multidisciplinary team, based on a comprehensive assessment of account tumor stage, liver function, ECOG performance status, vascular invasion, intrahepatic tumor burden, and patient preference.

Depending on the timing between locoregional and systemic therapy initiation, patients received either concurrent or sequential triple therapy. Among them, 41 patients (22.28% of the total cohort) underwent interventional therapy first, followed by sequential systemic treatment. The remaining 143 patients received concurrent triple therapy. All treatment regimens were according to the clinical guidelines in effect at the time, drug availability, and individual patient conditions, and were continued until disease progression, unacceptable drug toxicity, patient refusal to continue, or eligibility for conversion hepatectomy. Although the study spanned a long period, the basic principles of locoregional therapy, dose adjustment rules for systemic treatment, and imaging evaluation workflow at our center remained relatively consistent throughout the study period.

TACE delivers chemotherapeutic drugs followed by embolic agents directly into the artery that feeds the tumor, achieving high local drug concentrations and arterial embolization. The standard regimens include platinum drugs like cisplatin (60-80 mg/m^2) or oxaliplatin (100-150 mg/m^2) plus anthracyclines such as epirubicin (50-80 mg/m^2). The procedure was usually repeated every 3-6 weeks, with schedule adjustments according to the patient's overall status and their reaction to therapy.

HAIC differs from TACE: infuses chemotherapeutic drugs into liver tumors through an indwelling catheter, increasing local drug exposure while reducing systemic toxicity. In this study, the primary HAIC regimen was FOLFOX (oxaliplatin 85 mg/m^2, leucovorin 200 mg/m^2, fluorouracil 400 mg/m^2 loading dose followed by 2400 mg/m^2 continuous infusion). We repeated the procedure every 3-4 weeks. As with TACE, we adjusted schedules and doses based on each patient’s response and overall health.

1.2 Data Collection and Follow-up

We collected baseline clinical data and follow up information for all enrolled patients. Baseline data include the results of the last complete blood count (CBC) and biochemical tests prior to treatment, serum bilirubin, prealbumin, Eastern Cooperative Oncology Group (ECOG) performance status, ALBI grade, alpha-fetoprotein (AFP), hepatitis B virus (HBV) status, BCLC stage, CNLC stage, and Child-Pugh class. Based on CBC and biochemical tests results, we calculated the following indices: **NLR** was calculated as the ratio of neutrophil count ($\times 10^9$/L) to lymphocyte count ($\times 10^9$/L). **PLR** was defined as the pretreatment ratio of

peripheral platelet count to lymphocyte count: PLR = platelet count ($\times 10^9$/L) / lymphocyte count ($\times 10^9$/L).**GPR** was defined as the pretreatment ratio of serum gamma-glutamyl transferase to platelet count: GPR = GGT (U/L) / platelet count ($\times 10^9$/L).**GLR** was defined as the pretreatment ratio of serum gamma-glutamyl transferase to lymphocyte count: GLR = GGT (U/L) / lymphocyte count ($\times 10^9$/L).**AAR** was defined as the pretreatment ratio of serum aspartate aminotransferase to alanine aminotransferase: AAR = AST (U/L) / ALT (U/L).**GAR** was defined as the pretreatment ratio of serum gamma-glutamyl transferase to albumin: GAR = GGT (U/L) / albumin (g/L).**ALR** was defined as the pretreatment ratio of serum aspartate aminotransferase to lymphocyte count: ALR = AST (U/L) / lymphocyte count ($\times 10^9$/L). Because the distribution of ALR was non-normal and no established clinical cutoff exists, we used the cohort median as the threshold for group stratification. The median ALR value in this cohort was 31.9; accordingly, ALR < 31.9 was defined as the low-ALR group, and ALR ≥ 31.9 as the high-ALR group.

Patients were followed regularly through outpatient visits or telephone contact to monitor disease progression, recurrence, metastasis, and survival. OS was defined as the time from treatment initiation to death from any cause or last follow-up. For patients lost to follow-up, survival time was recorded at the last confirmed date known alive, and such observations were treated as censored. The final follow-up cutoff date was May 31, 2025.

1.3 Assessment and Handling of Missing Data

The completeness of all candidate variables and outcome data was evaluated. All 184 patients had follow-up information suitable for OS analysis. Data for all candidate variables, including sex, ALBI grade, ALR, and OS outcomes, were complete; therefore, no imputation for missing values was performed.

1.4 Variable Selection and Prognostic Model Development

Candidate variables were pre-selected based on previous studies, clinical relevance, and routine accessibility in clinical practice. Univariate Cox regression was first performed to assess the association between each candidate variable and OS. Given that 77 death events were observed in this study, variables meeting a significance threshold of $P < 0.05$ were fed into the LASSO Cox model to minimize overfitting. A 10-fold cross-validation procedure was performed in the LASSO Cox regression to select λ. The λ that yielded the minimum cross-validated error (λmin) was adopted as the final selection criterion. Variables with nonzero coefficients at λmin were subsequently included in a multivariate Cox proportional hazards model to further evaluate their independent prognostic value.

The final prognostic model was determined based on the multivariate Cox regression analysis, taking into account statistical significance, clinical interpretability, and model parsimony. Regression coefficients from the final model were calculated for each patient. The median risk score of the entire cohort was then stratified patients into low- and high-risk groups. Kaplan-Meier analysis was used to derive survival curves. Median follow-up time was estimated using the reverse Kaplan–Meier method, and the number and proportion of

censored cases were reported. The log-rank test was applied to compare OS between the risk groups. A prognostic nomogram was constructed based on the final Cox model.

1.5 Model Performance Evaluation and Internal Validation

Harrell's C-index and time-dependent AUC at 6, 12, and 24 months were used to evaluate model discrimination, with corresponding 95% confidence intervals. Calibration was evaluated with calibration curves at 6, 12, and 24 months, and the impact of missing data was corrected using the inverse probability weighting method in the calibration analysis at fixed time points.

Prediction error was quantified with the Brier score, where lower values indicate better predictive accuracy. Decision curve analysis (DCA) was performed to assess the clinical net benefit of the model across a range of threshold probabilities. For internal validation, the Bootstrap resampling method (1,000 iterations) was used to assess optimistic bias and obtain bias-corrected performance estimates. The stability of each predictor's effect in the final multivariate Cox model was also evaluated using the Bootstrap resampling results.

1.6 Multicollinearity and Proportional Hazards Assumption Testing

To assess correlations among predictors in the multivariate Cox model, multicollinearity was diagnosed using the variance inflation factor (VIF) or the generalized variance inflation factor (GVIF). For multicategorical variables, the adjusted GVIF (GVIF^(1/(2×Df))) was used for assessment. A VIF or adjusted GVIF >5 was considered indicative of potential multicollinearity. The proportional hazards assumption was evaluated using Schoenfeld residuals, which included tests for each predictor and an overall test. A P value >0.05 for both individual variable tests and the global test was interpreted as no major violation of the proportional hazards' assumption.

1.7 Model Comparison

To evaluate the predictive value of the developed prognostic model relative to existing HCC prognostic tools and inflammation-related indices, this study compared the final model with Child–Pugh grade, ALBI grade, PALBI score, NLR, PLR, SII, and ALR. A Cox proportional hazards model was built for each of these indicators, and the linear predictor from each model was extracted as a risk score. The C-index was used to measure the discriminative performance of the models, and time-dependent ROC curves were performed to calculate the AUC for predicting 6-, 12-, and 24-month OS.

1.8 Subgroup and Sensitivity Analyses

To further assess the stability of the risk scoring model across various clinical subgroups, this study conducted subgroup analyses based on enrollment time and treatment regimen. Patients were divided into early-enrollment and late-enrollment groups based on the median enrollment time, and also stratified by the specific treatment regimen they received. In each subgroup, the Cox proportional hazards model was used to assess the association between

risk scores or risk stratification and OS, and interaction tests were conducted to determine whether there was heterogeneity in the predictive effect of the model across different subgroups.

Given the long enrollment period in this study and the possibility that systemic treatment strategies may have changed over time, a sensitivity analysis adjusted for enrollment period was performed. The enrollment period was included as an adjustment variable in the Cox model to examine whether the association between the risk score and OS was influenced by the timing of enrollment. The robustness of the final model results was evaluated by comparing the HR, 95% CI, and P values for the risk score or risk group before and after adjustment.

Due to the limited number of patients with ALBI grade 3, the standard Cox model might yield unstable effect estimates. Therefore, we performed additional sensitivity analyses using Firth penalized Cox regression and ridge penalized Cox regression. Firth Cox regression was used to reduce estimation bias arising from small sample sizes or rare categories, whereas ridge Cox regression was applied to shrink the model coefficients. These sensitivity analyses included the same set of predictor variables as the final multivariate Cox model. The results of the ridge Cox regression are reported based on the λmin selected by cross-validation.

Supplementary Figure and legends

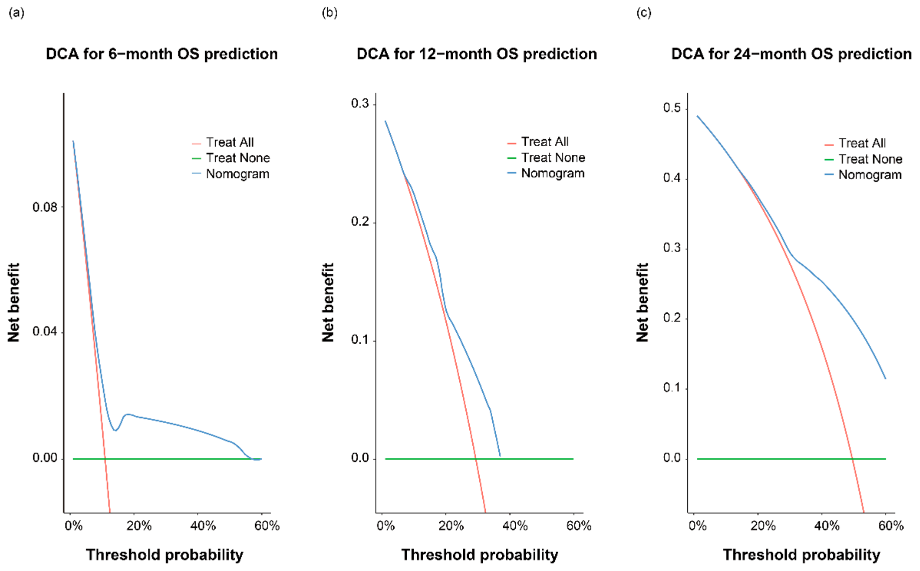

Supplementary Figure S1. Decision curve analysis (DCA) of the nomogram for overall survival prediction. Decision curve analyses were conducted for 6-month (**a**), 12-month (**b**), and 24-month (**c**) OS prediction. The nomogram demonstrated greater net benefit than the treat-all and treat-none strategies over selected ranges of threshold probabilities, with the most pronounced clinical utility observed for 24-month OS prediction.

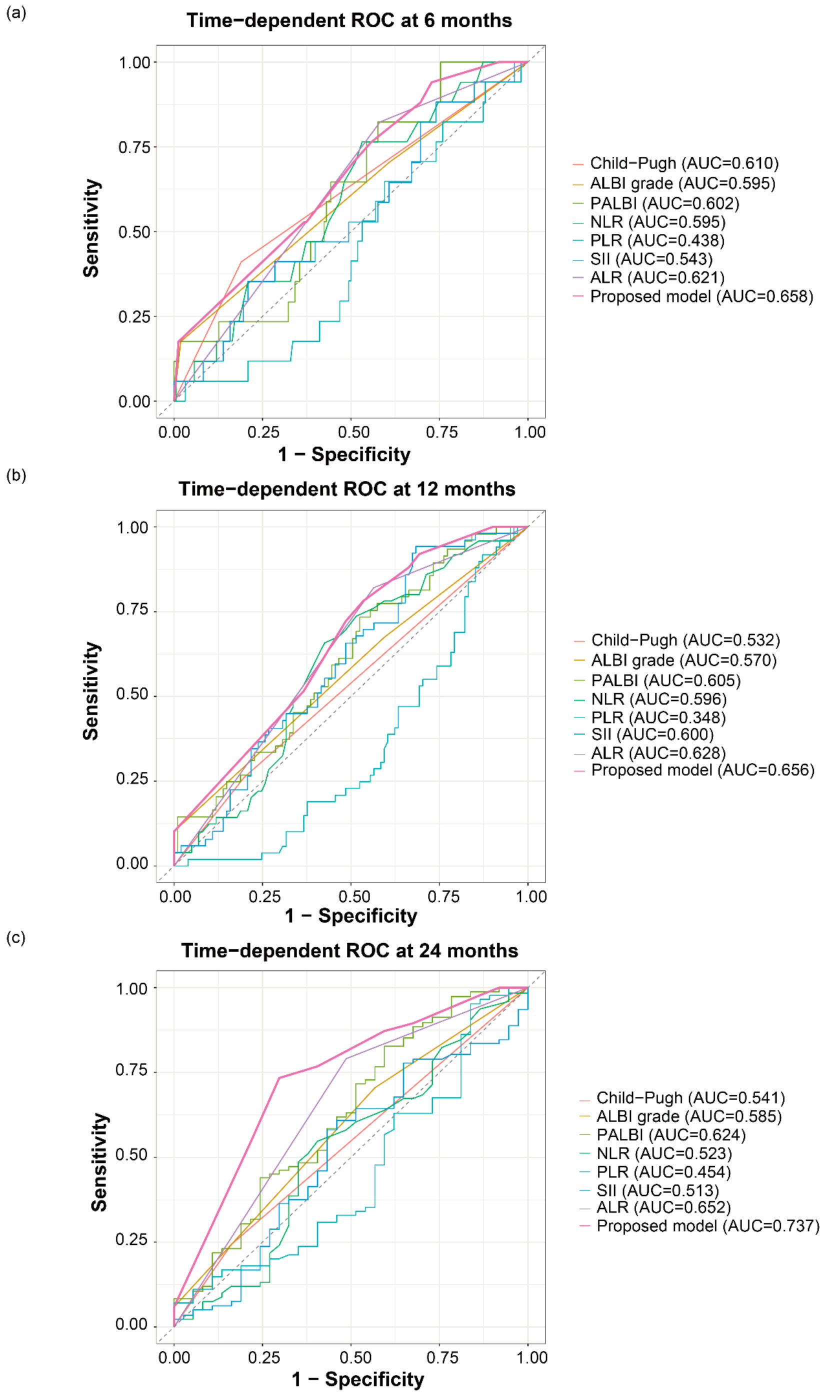

Supplementary Figure S2. Time-dependent ROC analysis of the model for overall survival prediction. Time-dependent ROC analysis was performed to evaluate the predictive performance of the proposed model with Child-Pugh grade, ALBI grade, PALBI score, NLR, PLR, SII, and ALR alone for 6-month (**a**), 12-month (**b**), and 24-month (**c**) overall survival. The proposed model demonstrated the highest area under the curve (AUC) at each time point.

Supplementary Table S1. Distribution of patients and death events according to ALBI grade.

ALBI grade	No. of patients	No. of events	Event rate
1	71	23	32.4%
2	107	49	45.8%
3	6	5	83.3%

Supplementary Table S2. Quantitative performance of the prognostic nomogram.

Metric	6 months	12 months	24 months
Time-dependent AUC	0.646	0.650	0.737
95% CI	0.530-0.762	0.565-0.735	0.638-0.835
Brier score	0.091	0.186	0.201
95% CI	0.057-0.124	0.156-0.216	0.171-0.230
Calibration intercept	-0.058	0.036	0.151
95% CI	-1.262-1.146	-0.501-0.573	-0.185-0.487
Calibration slope	0.973	1.027	1.323
95% CI	0.388-1.558	0.537-1.517	0.684-1.962
Number used for calibration	178	151	109

A calibration intercept of 0 and a calibration slope of 1 indicate perfect calibration. Lower Brier scores indicate lower prediction error.

Supplementary Table S3. Multicollinearity diagnostics and proportional hazards assumption tests for the final Cox model.

Variable	GVIF	Adjusted GVIF	PH test χ^2	PH test P value
Sex	1.010	1.005	0.939	0.33
ALBI grade	1.074	1.018	0.891	0.64
ALR	1.082	1.040	0.109	0.74
Global	—	—	2.119	0.71

Adjusted GVIF was calculated as GVIF^(1/(2×df)). An adjusted GVIF > 5 was considered indicative of potential multicollinearity. The proportional hazards assumption was assessed using Schoenfeld residuals.

Supplementary Table S4. Bootstrap sensitivity analysis of hazard ratio estimates in the multivariable Cox model.

Predictor	Original HR	Bootstrap median HR	Bootstrap 95% interval	Proportion of HR > 1
Male vs female	2.26	2.37	1.06-7.25	98.0%
ALBI grade 2 vs 1	1.09	1.07	0.64-1.93	60.2%
ALBI grade 3 vs 1	5.65	6.09	2.46-27.05	99.6%
High ALR vs low	2.38	2.40	1.38-4.97	99.7%

Bootstrap estimates were based on 1,000 resamples, and bootstrap 95% CIs were calculated using the percentile method. The proportion of HR > 1 represents the percentage of bootstrap resamples in which the estimated HR was greater than 1.

Supplementary Table S5. Sensitivity analyses using conventional Cox, Firth penalized Cox, and ridge Cox regression.

Variable	Conventional Cox HR	P value	Firth penalized Cox HR	P value	Ridge Cox HR using λmin
Male vs Female	2.26 (1.03-4.97)	0.042	2.12 (1.05-4.95)	0.034	1.77
ALBI grade 2 vs 1	1.09 (0.65-1.83)	0.739	1.08 (0.65-1.83)	0.765	1.09
ALBI grade 3 vs 1	5.65 (2.07-15.43)	0.0007	5.98 (2.05-14.76)	0.0022	4.37
ALR High vs Low	2.38 (1.37-4.15)	0.0022	2.33 (1.37-4.14)	0.0015	1.90

The conventional Cox and Firth penalized Cox models included sex, ALBI grade, and ALR as covariates. Female sex, ALBI grade 1, and the low ALR group were used as the reference categories. Ridge Cox estimates are reported using the λmin selected by cross-validation. Conventional 95% confidence intervals and P values are not available for the ridge Cox model.

Supplementary Table S6. Comparison of discriminative performance of the proposed model and established prognostic indicators.

Model	C-index	6-month AUC	12-month AUC	24-month AUC
Child-Pugh	0.540	0.610	0.532	0.541
ALBI grade	0.561	0.595	0.570	0.585
PALBI	0.579	0.602	0.605	0.624
NLR	0.563	0.595	0.596	0.523
PLR	0.410	0.438	0.348	0.454
SII	0.556	0.543	0.600	0.513
ALR alone	0.611	0.621	0.628	0.652
Proposed model	0.640	0.661	0.656	0.737

Supplementary Table S7. Sensitivity analysis adjusted for enrollment period.

Model	Variable	HR	95% CI	P value
Unadjusted model	High-risk vs low-risk	2.57	1.58-4.19	<0.001
Adjusted model	High-risk vs low-risk	2.57	1.58-4.18	<0.001
Adjusted model	Later vs earlier enrollment period	0.97	0.61-1.54	0.900

The adjusted model included risk group and enrollment period. Enrollment period was categorized according to the median enrollment date. The low-risk group and earlier enrollment period were used as reference categories.
